# Association between road accidents and low-grade hepatic encephalopathy among Sri Lankan drivers with cirrhosis: a prospective case control study

**DOI:** 10.1186/s13104-016-2106-3

**Published:** 2016-06-13

**Authors:** S. K. C. E. Subasinghe, Y. Nandamuni, S. Ranasinghe, M. A. Niriella, J. K. N. D. Miththinda, A. Dassanayake, A. P. de Silva, H. J. de Silva

**Affiliations:** Professorial Medical Unit, North Colombo Teaching Hospital, Ragama, Sri Lanka; Department of Medicine, Faculty of Medicine, University of Kelaniya, Thalagolla Road, PO Box 6, Ragama, GQ 11010 Sri Lanka; Department of Pharmacology, Faculty of Medicine, University of Kelaniya, Ragama, Sri Lanka

**Keywords:** Driving, Road accidents, Cirrhosis, Low-grade hepatic encephalopathy, Minimal hepatic encephalopathy, Psychometric tests

## Abstract

**Background:**

Low-grade hepatic encephalopathy (LGHE) comprises minimal hepatic encephalopathy (MHE) and grade 1 hepatic encephalopathy. LGHE has no or minimal recognizable symptoms but has mild cognitive and psychomotor deficits. Studies in Western countries have demonstrated increased road accidents (RA) among patients with MHE. Our objective was to investigate the association between Sri Lankan LGHE phenotype and RA.

**Study design and methods:**

A prospective, case–control study was conducted in the University Medical Unit, North Colombo Teaching Hospital, Ragama Sri Lanka. Patients with cirrhosis of any aetiology, without OHE, who had been driving during previous 1 month were included. A similar number of age matched, healthy control drivers were also enrolled. Both groups were subjected to five pencil-paper based psychometric tests used to detect LGHE in cirrhotics. Self-reported RA during the previous 1 month were recorded: categorized as ‘major’ when resulted in hospitalization of the involved, ‘minor’ when there were injuries, but not serious enough for hospitalization of the involved and ‘other’ when limited to damages to vehicle or environment without injuries.

**Results:**

Among 55 drivers with cirrhosis and LGHE [males, median age 53 years (range 30–60)], 7 (12.7 %) reported RA compared to 6 (10.9 %) among 55 controls [males; median age 51 years (range 30–60)]. There were no ‘major’ accidents in either group. 2/55 (3.6 %) cases and 2/55 (3.6 %) controls reported ‘minor’ accidents.

**Conclusion:**

There was no increased frequency of RA among Sri Lankan drivers with LGHE compared to healthy controls. This is with the limitation of the study based only on self reported RA.

## Background

Road accidents (RA) are the leading cause of hospital admissions in Sri Lanka [1]. Although a number of factors contribute to the occurrence of RA, human factors are one of the most cited causative influences [2, 3]. Minimal hepatic encephalopathy (MHE) is a complex neuro-psychological complication of cirrhosis, which is characterized by delayed reaction time and abnormal response inhibition [4–6]. MHE represents a part of the “low-grade” or “covert” hepatic encephalopathy (LGHE) in its mildest form. LGHE can be defined as the presence of test-dependent or clinical signs of brain dysfunction in patients with cirrhosis who are not disoriented or display asterixis. The term “minimal” conveys that there is no clinical sign, cognitive or other, of hepatic encephalopathy. The term “low-grade” includes MHE and grade 1 hepatic encephalopathy. [7].

Clinically, patients with MHE and LGHE appear to be free of neuropsychiatric abnormalities, but exhibit specific, reversible, quantifiable neuropsychiatric and electroencephalographic abnormalities [8–11]. The prevalence of MHE in cirrhosis has been reported to vary from 30 to 84 %, depending on both the tests used and population studied [12, 13]. The diagnosis of MHE and LGHE is only through specialized neuro-psychometric or neuro-physiologic tests because there are no readily identifiable clinical manifestations [7, 14].

MHE and LGHE can have a significant impact on quality of life, ability to function in daily life and progression to overt hepatic encephalopathy (OHE) [15–17]. The ability to drive a car can be impaired in patients with MHE and it is a strong predictor of traffic violations and motor vehicle accidents in a self-reported setting [18]. However, studies on driving which largely relied upon the judgment of a designated driving instructor, showed conflicting results. One study showed patients with cirrhosis had no significant differences in their performance on a simulator or during actual driving conditions when compared to matched controls. In this study, stable subjects with cirrhosis and evidence of portal hypertension, portal-systemic shunting, abnormal neuropsychological tests and no prior history of overt encephalopathy did not exhibit a major impairment in their fitness to drive [19]. Another study showed similar findings that the presence of MHE did not necessarily predict driving unfitness. However, despite significant driving deficits, patients with overestimated their driving abilities. It also showed that computer-based testing cannot reliably predict driving fitness [20]. Conversely, another prospective controlled study revealed fitness to drive a car to be impaired in the setting of MHE [21]. Thus, it was recommended that patients with liver cirrhosis be tested for MHE, informed in case of abnormal test results and possible therapy known to improve psychometric test be initiated among them if continuing to drive. Furthermore, patients with cirrhosis and MHE diagnosed using inhibitory control test (ICT) have a significantly higher crash rate over the preceding year and on prospective follow-up compared to patients without MHE [22].

These is limited data on this topic from the South Asian region. The objective of the present study was to investigate above association of possible increase of RA and suitability to continue to drive among a cohort of Sri Lankan drivers with cirrhosis and LGHE.

## Methods

This was a case control study. Data was collected prospectively. Taking into consideration the reported difference in the accident rate between those with and without MHE in the literature, our study was adequately powered to detected difference of 20 % of RA among patients with cirrhosis and LGHE compared to controls. Informed written consent was obtained from both case and controls prior to enrollment after thorough explanation of the purpose and nature of the study. Fifty-five patients with cirrhosis and LGHE (MHE and grade 1 hepatic encephalopathy), who were regularly driving, were recruited from the Gastroenterology Clinic, University Medical Unit, Colombo North Teaching Hospital, Ragama, Sri Lanka, during August 2013 to November 2014, by convenience sampling. Fifty-five age and sex matched healthy drivers who were teetotalers were recruited as controls. All patients and controls who were recruited had a valid driving license issued from the Department of Motor Traffic, Sri Lanka.

The diagnosis of cirrhosis was based on clinical, biochemical and ultrasonographic findings that were compatible with cirrhosis. Exclusion criteria were OHE or a history of OHE, recent history of alcohol intake (within the past 1 month), use of medications precipitating HE, history of hepatocellular carcinoma, organ failure, neurological or psychiatric disorders, visual, hearing disorders or myopathies that may influence ability to drive and complete the psychometric tests.

A questionnaire was completed by each driver. It included demographic data, alcohol intake (amount and type), residential area (rural/urban), education level, aetiology and duration of cirrhosis and history of cirrhotic complications in the past. Similarly, specific driving details were also recorded. It included whether the person has obtained a valid driving license from relevant authority and the duration, types of vehicles driven, average distance per week and average time duration of driving per day. Self-reported RA during the previous 1 month were recorded and were categorized. Accidents were considered as ‘major’ when the injured person/s involved needed hospital admission, and as ‘minor’ when the injured person/s involved did not require hospital admission. Accidents were categorized as ‘other’ when there were no injuries to person/s but included damage to the vehicle or the environment. Basic haematological and biochemical investigation results were also recorded in order to determine child-turcott-pugh (CTP) class and the MELD scores.

A standard psychometric test battery [number connection test A and B (NCT A and B)], line tracing test (LTT), digit symbol test (DST) and serial dotting test (SDT)] was administered to both patients and controls by trained research assistants and the time to complete NCT A and B and SDT tests including error correction times were recorded. In DST, subjects were asked to substitute the symbols to 80 numbers as in the standard test in 90 s and the number of properly substituted numbers were measured. In LTT, time taken for completion of the test and number of errors were recorded.

Before administering the above psychometric testing to cirrhotic drivers, their mental state was assessed by using a validated Sri Lankan version of Montreal cognitive assessment scale (MOCA) [23]. An initial demonstration was performed for each participant so as to be familiar with the test. A longer time to complete each test with error correction was considered a poor test performance in NCT A/B, SDT and LTT. Additionally, number of errors also taken into account in LTT. Number of correctly substituted symbols within 90 s was taken in DST; a performance impaired beyond 2 standard deviations (2SD) of the control performance towards the pathological direction in any 3 or more tests was considered as diagnostic of LGHE.

Ethical approval for the study was obtained from the Ethics Review Committee, Faculty of Medicine, University of kelaniya, Ragama, Sri Lanka.

## Results

Patients and controls are very well adjusted with regard to age, sex, education, residence and driving details. Fifty-five cirrhotic patients with LGHE and 55 age and sex matched controls who were teetotalers were recruited. They were all males as only male cirrhotic drivers with LGHE could be identified at screening. The demographic characteristics and the types of accidents of the studied groups are shown in Table 1. Majority (n = 45, 82 %) of the patients were in 51–60 years of age. Therefore in the whole group, the normal reference ranges/means for the psychometric tests were almost similar to the values of 51–60 years group.

**Table 1:** Demographic characteristics of the screened drivers

Parameter	LGHE group (n = 55)	Control group (n = 55)
Age (mean years ± SD)	51.4 ± 8.4	49.5 ± 7.5
30–40 years	3	3
41–50 years	7	7
51–60 years	45	45
Level of education		
<Grade 10	21	19
Secondary	24	23
Tertiary/university	10	13
Residence		
Urban	43	45
Rural	12	10
Duration of the license		
<5 years	08	10
5–10 years	21	20
>10 years	26	25
Types of vehicles		
Bicycles/motor bikes	15	16
Cars/vans	20	22
Passenger services	08	10
Mixed	12	07
Distance of driving per week		
<50 km	28	29
>50 km	27	26
Duration of driving per day		
<1 h	19	20
>1 h	36	35
Types of accidents		
With people	2	2
With another vehicle	3	3
With environment	2	1

None of 110 persons in the study had evidence of cognitive impairment, and all had a score of more than 25 in the Sri Lankan version of the MOCA. The clinical and biochemical characteristics of the cirrhotic group are demonstrated in Table 2. 54.5 % of patients had cryptogenic cirrhosis and 56.3 % were categorized as Child class A. The presence of gastro-oesophageal varices was the only complication encountered in the MHE group (40 %). The findings of individual psychometric tests are given in Table 3. Apart from the LTT, which is a relatively easy test to perform with minimal errors and error correction time, the performance in all other tests was significantly impaired in the LGHE group compared to controls.

**Table 2:** Clinical and biochemical characteristics of the cirrhotic (LGHE) group

Parameter	n (55)
Aetiology of cirrhosis	
Alcohol	16
Cryptogenic	30
NASH	03
Other	06
Child-pugh grade	
Child A	31
Child B	18
Child C	06
Duration since diagnosis of cirrhosis	
<1 month	27
1–3 months	20
>3 months	08
Cirrhotic complications	
Gastro-oesophagealvarices	22
SBP	None
OHE	None
Porto-systemic shunts	None
HRS/AKI	None
HCC	None
Sodium (mmol/l)	136.9 ± 3.9
Potassium (mmol/l)	4.7 ± 4.9
ALT (IU/l)	54.6 ± 35.1
AST (IU/l)	69.5 ± 41.5
Albumin (g/l)	3.5 ± 0.6
Bilirubin (mg/dl)	1.4 ± 0.8
INR	1.7 ± 0.35
Haemoglobin (g/dl)	11.2 ± 2.1
Platelet count	105 ± 41
Creatinine (mg/dl)	1.1 ± 0.3

*SBP* spontaneous bacterial peritonitis, *OHE* overt hepatic encephalopathy, *HRS* hepato- renal syndrome, *AKI* acute kidney injury, *HCC* hepatocellular carcinoma, *ALT* alanine transaminase, *AST* aspartate transaminase, *INR* International normalized ratio

**Table 3:** Outcome of psychometric tests

Psychometric test	Mean	Standard deviation	P value*
NCT-A			
LGHE	83.76	15.85	*<0.001*
Controls	57.60	12.28	
NCT-B			
LGHE	243.02	57.99	*<0.001*
Controls	89.33	13.74	
SDT			
LGHE	82.62	10.96	*<0.001*
Controls	58.75	6.46	
LTT			
LGHE			
Time for completion	80.84	11.08	0.59
Number of errors	7.23	4.21	0.49
Controls			
Time for completion	82.29	17.21	
Number of errors	6.41	4.01	
DST			
LGHE	34.28	17.85	*<0.001*
Controls	67.25	8.25	

Italics indicate statistically significant P value

*NCT* number connection test, *SDT* serial dotting test, *LTT* line tracing test, *DST* digit symbol test, *LGHE* low-grade hepatic encephalopathy

* Chi square test of significance

Among 55 cirrhotic drivers with LGHE, 7 (12.7 %) reported accidents compared to 6 (10.9 %) among the 55 controls (p 0.38; Chi square test). There were no ‘major’ accidents in either group. 2/55 (3.6 %) cases and 2/55 (3.6 %) controls reported ‘minor’ accidents (p 0.69; Fisher’s exact test). The two minor accidents in each group had resulted in mild abrasions. ‘Other’ accidents were various types of events causing some damage to the vehicle or environment.

## Discussion

Impaired attention, visuo-motor coordination, psychomotor speed and response inhibition among patients with cirrhosis and MHE or LGHE may explain the previously reported increased incidence of RA [24]. However, the present study failed to demonstrate increased RA among a cohort of Sri Lankan drivers with cirrhosis and LGHE compared to an age matched healthy control group. Our finding is at variance with the findings of some studies conducted in Western countries.

In a previous study, patients with MHE had impaired driving skills and possible association of MHE with motor vehicle crashes [22]. That study used standard psychometric tests (SPT) or inhibitory control test (ICT) to diagnose MHE. A significantly higher proportion of MHE patients diagnosed using ICT had motor vehicle crashes over the preceding year compared to those without MHE (17 vs. 0 %). Interestingly, SPT did not differentiate between those with/without a history of crashes [22]. Furthermore, patients with MHE followed up prospectively were found to have significant future violations and crashes compared to those without MHE (22 vs. 7 %). This finding was also observed only in the MHE group diagnosed using ICT [25]. Another study demonstrated that cirrhotics with MHE had slightly higher traffic violations and accidents (21 vs. 17 % within 1 year and 36 vs. 33 % within 5 years respectively) compared to those without MHE [18]. Studies using on-road driving tests have demonstrated that MHE patients have significant defects in reaction time, car handling, adaptation, cautiousness and maneuvering [21, 25]. Defects in navigation skills and a higher rate of illegal turns in such patients render them unsafe drivers [26, 27].

Therefore, from the available evidence from Western studies, a diagnosis of MHE or LGHE does not automatically mean that the affected subject is a dangerous driver [7, 28]. Medical providers are not trained to formally evaluate fitness to drive. However, doctors cannot evade the responsibility of counseling patients with LGHE on the possible dangerous consequences of their driving. The safest advice is to stop driving until the responsible driving authorities have formally cleared the patient for safe driving [7].

Among the specialized tests to detect MHE or LGHE, neurophysiological tests such as critical flicker frequency (CFF) is fast and easy to administer and is recommended by some [28]. However, in Sri Lanka only psychometric tests can be performed in epidemiological studies at present since neurophysiological tests such as CFF is not readily available in clinical practice.

The Porto-Systemic Encephalopathy syndrome test (PSE-syndrome test) used by Weissenborn et al., has been validated for MHE diagnosis in Germany, Italy and Spain [14]. This test is recommended for diagnosing MHE and provides the PHES (psychometric hepatic encephalopathy score). However, this has not been validated for the other countries including Sri Lanka. Therefore, we decided to use our own criteria to detect MHE.

Absence of female cirrhotic drivers participation for this study is a major drawback and this is mainly due to the cultural background and overall low number of female drivers compared to the males in the country. After assurance of no impact on current driving license, irrespective of the test result outcome, none of the participants declined to participate. The study was only based on self-reported RA. This was a limitation of our study. We considered RA during a relatively short period (previous 1 month) in order to minimize the events possibly forgotten.

The present study failed to show an increase in the incidence of RA in a cohort of Sri Lankan drivers with cirrhosis and LGHE compared to healthy controls. Our study was adequately powered to detected difference of 20 % of RA among LGHE patients compared to controls. The absence of driving or riding on high speed motorways may at least partly explain this lack of increased RA in the study group. Computer based ICT, driving stimulation tests and on-road driving tests could be used in a large cohort to further evaluate LGHE and its impact on driving. If such tests also fail to demonstrate impairment of driving skills, this will validate the findings of the present study. Furthermore, the finding of this study has to be interpreted with the limitation of the short period of self-reported RA among the Sri Lankan LGHE phenotype.

In conclusion, the findings of this study suggest that driving may be safe among patients with LGHE, but without OHE. Although our finding should be considered in a local (Sri Lankan) context, it will have practical implications with regards to advice on driving, holding a valid driving license and continuing to drive for patients with cirrhosis and LGHE.

## Abbreviations

RA: road accidents
MHE: minimal hepatic encephalopathy
LGHE: low-grade hepatic encephalopathy
OHE: overt hepatic encephalopathy
ICT: inhibitory control test
CTP: child-turcott-pugh
NCT A and B: number connection test A and B
LTT: line tracing test
DST: digit symbol test
SDT: serial dotting test
MOCA: Montreal cognitive assessment scale
SPT: standard psychometric tests
ICT: inhibitory control test
